# Overexpression of miR-1306-5p, miR-3195, and miR-3914 Inhibits Ameloblast Differentiation through Suppression of Genes Associated with Human Amelogenesis Imperfecta

**DOI:** 10.3390/ijms22042202

**Published:** 2021-02-23

**Authors:** Hiroki Yoshioka, Yin-Ying Wang, Akiko Suzuki, Meysam Shayegh, Mona V. Gajera, Zhongming Zhao, Junichi Iwata

**Affiliations:** 1 Department of Diagnostic & Biomedical Sciences, School of Dentistry, The University of Texas Health Science Center at Houston, Houston, TX 77054, USA; Hiroki.Yoshioka@uth.tmc.edu (H.Y.); akikosuz925@gmail.com (A.S.); meysamshayegh@yahoo.com (M.S.); gajeramona@gmail.com (M.V.G.); 2 Center for Craniofacial Research, The University of Texas Health Science Center at Houston, Houston, TX 77054, USA; 3 Center for Precision Health, School of Biomedical Informatics, The University of Texas Health Science Center at Houston, Houston, TX 77030, USA; yingxiao8958@gmail.com; 4 Human Genetics Center, School of Public Health, The University of Texas Health Science Center at Houston, Houston, TX 77030, USA; 5 MD Anderson Cancer Center UTHealth Graduate School of Biomedical Sciences, Houston, TX 77030, USA

**Keywords:** microRNAs, tooth formation, amelogenesis imperfecta, enamel formation, tooth development

## Abstract

Amelogenesis imperfecta is a congenital form of enamel hypoplasia. Although a number of genetic mutations have been reported in humans, the regulatory network of these genes remains mostly unclear. To identify signatures of biological pathways in amelogenesis imperfecta, we conducted bioinformatic analyses on genes associated with the condition in humans. Through an extensive search of the main biomedical databases, we found 56 genes in which mutations and/or association/linkage were reported in individuals with amelogenesis imperfecta. These candidate genes were further grouped by function, pathway, protein–protein interaction, and tissue-specific expression patterns using various bioinformatic tools. The bioinformatic analyses highlighted a group of genes essential for extracellular matrix formation. Furthermore, advanced bioinformatic analyses for microRNAs (miRNAs), which are short non-coding RNAs that suppress target genes at the post-transcriptional level, predicted 37 candidates that may be involved in amelogenesis imperfecta. To validate the miRNA–gene regulation association, we analyzed the target gene expression of the top seven candidate miRNAs: miR-3195, miR-382-5p, miR-1306-5p, miR-4683, miR-6716-3p, miR-3914, and miR-3935. Among them, miR-1306-5p, miR-3195, and miR-3914 were confirmed to regulate ameloblast differentiation through the regulation of genes associated with amelogenesis imperfecta in AM-1 cells, a human ameloblastoma cell line. Taken together, our study suggests a potential role for miRNAs in amelogenesis imperfecta.

## 1. Introduction

Dental enamel is the most hardened mineralized tissue in the body, composed of 95% of hydroxyapatite crystals (mainly calcium and phosphate, but also magnesium, potassium, fluoride, and sodium), with the remaining consisting of matrix proteins and water [1,2,3]. Ameloblasts secrete an enamel matrix during amelogenesis, which comprises a pre-secretory (inductive), secretory, and maturation stage [1,4,5]. During the pre-secretory stage, inner enamel epithelial cells on the dentin matrix differentiate into ameloblasts. At the subsequent secretory stage, polarized ameloblasts with Tomes’ process start to secrete enamel matrix proteins such as ameloblastin (AMBN), amelogenin (AMELX), amelotin (AMTN), and enamelin (ENAM). These enamel matrix proteins are then phosphorylated by extracellular serine/threonine protein kinase FAM20C and cleaved by metallopeptidase 20 (MMP20), and bind to calcium ions, forming primary enamel crystals [6,7,8,9,10]. Finally, at the maturation stage, ameloblasts differentiate into ruffle-ended (RA) and smooth-ended (SA) ameloblasts. At the stage of RA and SA ameloblast differentiation, the degradation and removal of the enamel matrix from primary enamel crystals by kallikrein-4 (KLK4), and the increase in hydroxyapatite concentration, induce the growth of highly mineralized enamel crystals. During the maturation stage, extracellular pH changes from acidic to neutral, which induces calcium and phosphate ion deposition and enamel crystal nucleation in RA and enamel crystal growth in SA, respectively. This acid–base balance of extracellular pH is regulated by bicarbonate and hydrogen ion synthesis and transportation between ameloblasts and primary enamel [11,12,13,14]. Any failure in amelogenesis leads to absence or poor development of tooth enamel, a.k.a. amelogenesis imperfecta, a congenital tooth defect that affects the highly mineralized enamel of primary and permanent teeth [15], resulting in a high incidence of severe dental caries, pain, early tooth loss, and remarkably decreased quality of life (e.g., poor appearance, ingestion problems, and discomfort).

Amelogenesis imperfecta is either syndromic or non-syndromic and may be X-linked, autosomal recessive, or autosomal dominant. Patients with amelogenesis imperfecta suffer from tooth pain and compromised aesthetic appearance, which negatively affect quality of life [16]. While a couple of classifications for amelogenesis imperfecta have been proposed according to its clinical features and inheritance patterns [17], the classification by Witkop (1988) is the most widely used. In this classification, amelogenesis imperfecta is categorized into four major types: hypoplastic (type I), hypomaturation (type II), hypocalcified (type III), and hypomature/hypoplastic enamel with taurodontism (type IV). These four types are further classified into 17 subtypes, based on the causative gene mutations and inheritance patterns [17,18]. Hypoplastic amelogenesis imperfecta (type I) is caused by defects in ameloblasts at the secretory stage of amelogenesis, with varying degree of severity, ranging from thin mineralized enamel to complete absence of enamel, but with an enamel radiodensity still higher than that of dentine. In hypomaturation amelogenesis imperfecta (type II), the enamel is soft and easily chipped, caused by a failure in the maturation of the enamel crystal structure due to incomplete removal of the enamel matrix. While initial enamel formation and calcification normally occur in type II, the radiodensity is similar to that of dentine. Hypocalcified amelogenesis imperfecta (type III), in which the enamel shows less calcification due to a failure in calcium-ion transportation to the maturating enamel, is characterized by a rough, soft enamel surface with standard thickness, but lower radiodensity versus dentine. Lastly, the most severe form, amelogenesis imperfecta type IV, is characterized by the co-occurrence of taurodontism in molars. These classifications are becoming obsolete with the advances made in genetic diagnosis, since phenotypes often present as a mix of the different phenotypic forms. Up to now, genome-wide association studies (GWAS) and linkage studies have identified genetic susceptibility to amelogenesis imperfecta in various populations and ethnic groups (e.g., 43 per 10,000 in Turkey, 13 per 10,000 in Sweden, and 1 per 14,000 in the US, with a worldwide prevalence of 0.5% [19,20]). There are several databases for amelogenesis imperfecta. Examples include the LOVD amelogenesis imperfecta database (http://dna2.leeds.ac.uk/LOVD/), the Genetic and Rare Diseases Information Center (GARD) from the National Institutes of Health (NIH) (https://rarediseases.info.nih.gov/diseases/5791/amelogenesis-imperfecta), and National Organization for Rare Disorders (NORD) (https://rarediseases.org/rare-diseases/amelogenesis-imperfecta/) (all the websites here were lastly accessed on 13 January 2021). However, the number of novel gene mutations associated with amelogenesis imperfecta identified has been growing recently.

MicroRNAs (miRNAs) are short non-coding RNAs, which are post-transcriptional regulators of target genes [21]. The expression of miRNAs is regulated epigenetically or genetically; therefore, it is considered to be a target of environmental factors that influence gene expression. Pri-miRNAs are transcribed from their coding regions, and then converted into pre-miRNAs by DROSHA, a ribonuclease III enzyme. The pre-miRNAs are finally converted to mature miRNAs by DICER, an RNase III endonuclease. Each miRNA binds to the recognition site on the 3′ untranslated region (3′ UTR) in multiple genes; therefore, each miRNA can target multiple genes and each gene’s expression is regulated by multiple miRNAs [22]. Thus, the regulatory network of miRNA–gene is complex, and miRNAs and their clusters are involved in embryonic development and pathogenesis/prognosis through spatiotemporal expression [23,24]. Involvement of miRNAs in amelogenesis and tooth development has been suggested by miRNA microarray analysis, overexpression analysis in mouse ameloblast-like LS-8 cells, and knockout mice (Supplementary Table S1). Currently, more than 1000 miRNAs are known to be specifically expressed in enamel-containing structures. Among them, miR-153 plays roles in enamel protein endocytosis and lysosomal degradation [25,26]. *Dicer1* knockout mice fail to generate mature miRNAs, and mice with an epithelial-specific deletion of *Dicer1* (*Pitx2-Cre;Dicer1* and *K14-Cre;Dicer1* conditional knockout mice) exhibit hypoplastic or lack of enamel formation [27,28]. In addition, miR-214 null mice exhibit hypomineralized enamel through suppression of *Tgfb1* and *Clu* expression in the tooth germ [29,30]. Taken together, these data indicate that the expression of genes associated with amelogenesis imperfecta may be regulated by miRNAs during amelogenesis.

In this study, in order to identify causative regulatory pathways within the complex etiology of amelogenesis imperfecta, we conducted a literature search to generate a list of genes associated with amelogenesis imperfecta and performed bioinformatic analysis on genes associated with human amelogenesis imperfecta. We experimentally tested the regulation of the predicted candidate genes and miRNAs, using human ameloblastoma AM-1 cells. Thus, this study aims to understand the genetic susceptibility to amelogenesis imperfecta and the specific role of miRNAs in the disease in humans.

## 2. Results

### 2.1. Literature Search

**Figure 1:**
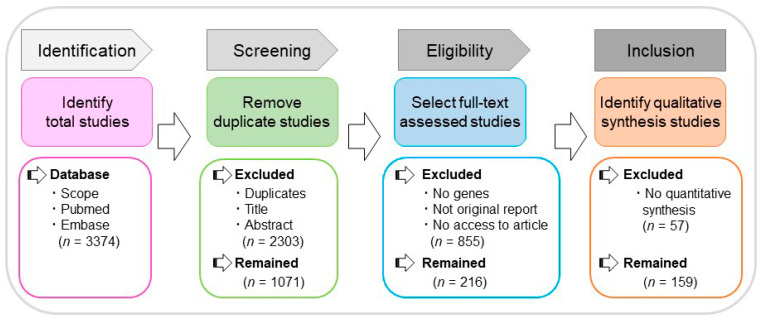
The flowchart for literature mining based on the Preferred Reporting Items for Systematic Reviews and Meta-Analyses (PRISMA) guideline, including the sequential steps for the identification, screening, eligibility check, and qualification of the literature.

Figure 1 depicts the flowchart for literature mining based on the Preferred Reporting Items for Systematic Reviews and Meta-Analyses (PRISMA). Our systematic search identified a total of 3374 publications in April 2019. After eliminating 2088 duplicates, the remaining 1286 articles were further screened using titles and abstracts, which resulted in 1071 publications being further excluded based on the exclusion criteria. The remaining 216 articles were assessed for eligibility by manual review of the full text. Through this process, 159 articles satisfying all the criteria were selected, while 56 articles were excluded. The selected 159 studies were used to identify genes associated with amelogenesis imperfecta. To retrieve articles that were not identified through the systematic review, we also conducted a manual search based on the review articles and original articles related to tooth defects.

Through the systematic literature review and manual search, a total of 56 candidate genes for amelogenesis imperfecta were identified: 39 genes through the systematic review and 17 genes through the manual search (Supplementary Tables S2, S3.1 and S3.2). Mutations in 15 genes were reported in non-syndromic cases of amelogenesis imperfecta (17/56 = 30.3%), while mutations in the remaining 39 genes were reported in syndromic cases (39/56 = 68.4%). Mutations in *AMELX*, *DLX3*, *LAMA3*, *LAMB3,* and *WDR72* were reported in both non-syndromic and syndromic amelogenesis imperfecta (Table 1). According to the classification of the condition, there were 40 genes for the autosomal recessive type, 16 genes for the autosomal dominant type, five genes for the X-linked type (recessive and dominant), and six genes for both the autosomal recessive and dominant types (Table 2). The hypoplastic type (type I) with mutations in *ENAM* was the most frequent, whereas type IV was less frequent and associated with mutations in *ALPL*, *DLX3,* and *LAMB3* (Table 3 and Supplementary Table S4). Of note, several genes have been reported for different types or mixed types of amelogenesis imperfecta. In addition, while amelogenesis imperfecta is reported as one of the clinical features (in some cases) in 19 syndromes, genetic analysis has not been conducted in patients with this condition only. Besides amelogenesis imperfecta, enamel hypoplasia is reported as one of the clinical aspects in 71 syndromes (Supplementary Tables S3.3, S3.4, S3.5, S5, and S6).

### 2.2. Bioinformatic Analysis

**Figure 2:**
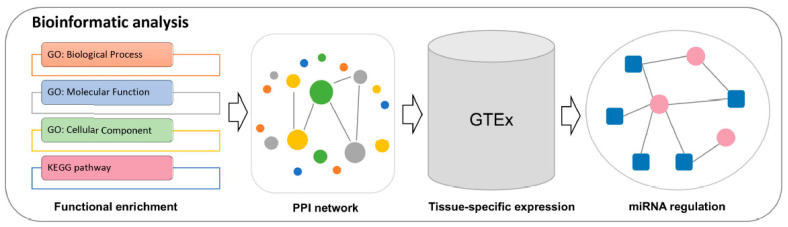
Bioinformatic analysis of the identified amelogenesis imperfecta-related genes, including functional enrichment analysis, construction of amelogenesis imperfecta-related protein interaction network, tissue-specific expression of amelogenesis imperfecta-related genes, and construction of miRNA–gene regulations.

To identify the role and the regulatory mechanism of each amelogenesis imperfecta-related gene, we conducted bioinformatic analyses for functional enrichment, protein‒protein interactions (PPIs), tissue-specific expression, and miRNA–gene regulation using the 56 genes associated with the condition (Figure 2).

**Figure 3:**
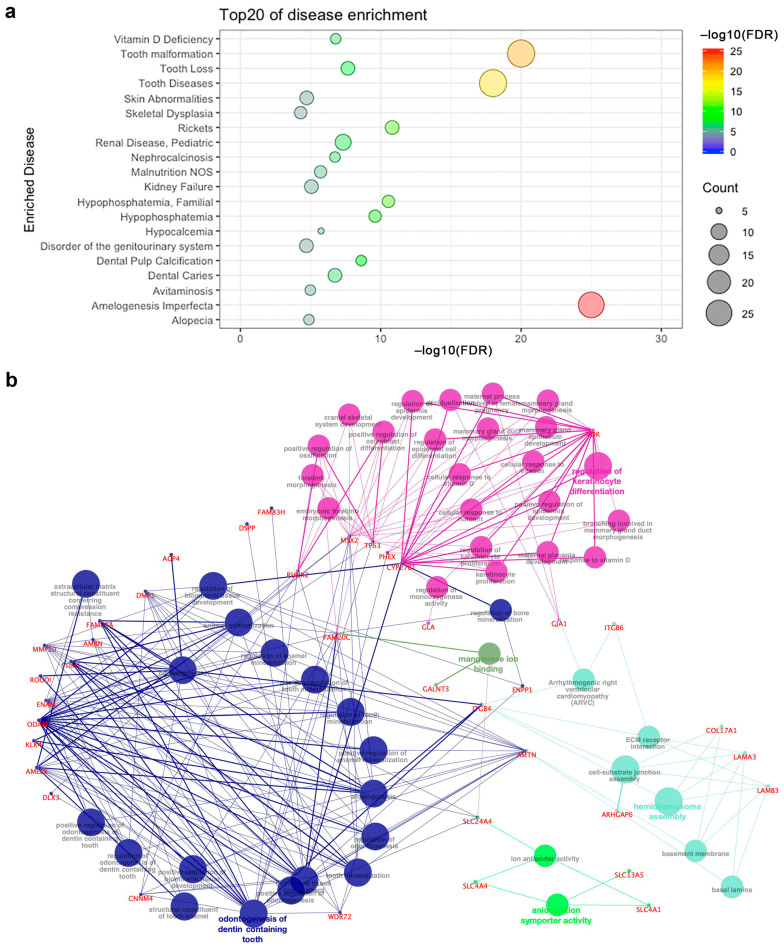
Functional enrichment analysis of amelogenesis imperfecta-related genes. (**a**) Top 20 most significantly enriched diseases. (**b**) Enriched pathway–gene network: nodes annotated in black represent pathways, while the others in red denote genes. Genes associated with multiple terms, especially multiple categories of functions, are marked with different colors.

First, we performed functional enrichment analysis for genes associated with amelogenesis imperfecta using Webgestalt (Figure 3a). Here, only enriched terms with adjusted *p*-value (FDR) < 0.05 were selected. We confirmed that the genes were significantly enriched in the category “amelogenesis imperfecta” (adjusted *p*-value = 1.22 × 10^−25^) as well as “tooth diseases” (adjusted *p*-value = 2.14 × 10^−18^) and “tooth malformation” (adjusted *p*-value = 3.60 × 10^−22^). These genes were also associated with pediatric diseases, bone diseases, and epithelial tissue diseases, including pediatric renal disease (adjusted *p*-value = 4.80 × 10^−8^), vitamin D deficiency (adjusted *p*-value = 1.63 × 10^−7^), rickets (adjusted *p*-value = 1.54 × 10^−11^), and skin abnormalities (adjusted *p*-value = 1.88 × 10^−5^) (Supplementary Table S7). We also confirmed that the GO terms were enriched in amelogenesis imperfecta-related terms, tooth mineralization (GO: 0031012) (adjusted *p*-value = 0), and odontogenesis (GO: 0042476) (adjusted *p*-value = 0) (Supplementary Table S8). Among the Kyoto Encyclopedia of Genes and Genomes (KEGG) canonical pathways, only the term “extracellular matrix (ECM)–receptor interaction” was uniquely and significantly enriched with the *ITGB6*, *ITGB4*, *LAMA3*, and *LAMB3* genes (adjusted *p*-value = 1.22 × 10^−2^) (Supplementary Table S8).

To further interpret the role of differentially expressed genes (DEGs) in the enriched pathways, we used the plugin model ClueGO (v. 2.5.7) in Cytoscape (v. 3.6.0) to construct an enriched pathway–gene network. In this network, the pathway with adjusted *p*-value < 0.05 was selected, and genes involved in more than two pathways were kept. Genes associated with multiple terms, especially multiple categories of functions, may play an essential role in the pathogenesis of amelogenesis imperfecta. Most of the genes were enriched in multiple terms of the same categories with similar functions (marked with the same color in Figure 3b): regulation of keratinocyte differentiation (GO: 0045616; red), and odontogenesis of dentin-containing (GO: 0042475; blue). By contrast, *AMTN*, *FAM20C*, *ITGB4*, *RUNX2,* and *SLC24A4* were involved in multiple function categories (Figure 3b; Supplementary Table S8). Thus, the functional enrichment analysis suggests several common mechanisms for amelogenesis imperfecta and common mechanisms for enamel and dentin defects. While we excluded genes which, when mutated, cause dentin anomalies and secondary defects in enamel, there is the possibility of having some minor dentin defects in some cases.

**Figure 4:**
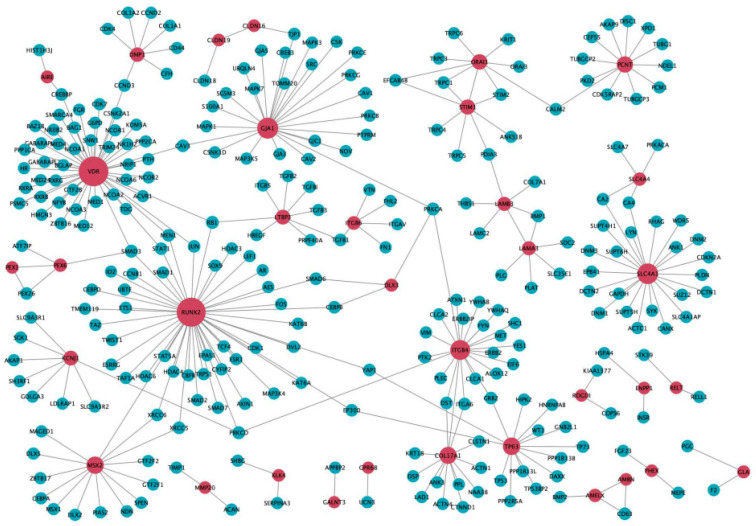
Protein–protein interaction (PPI) network of amelogenesis imperfecta-related genes. Red nodes represent amelogenesis imperfecta-related genes and blue nodes denote the genes that interact with amelogenesis imperfecta genes in the PPI network. The size of the nodes was ranked according to node degree. The PPI network we used here was a combination of all five databases.

Various types of mutations have been identified in diseases with complex etiology [19]. To identify the affected biological processes in amelogenesis imperfecta, we investigated PPIs using the combination of all five databases: HPRD [31], BioGrid [32], IntAct [33], MINT [34], and DIP [35]. We found that *VDR*, a vitamin D receptor in which mutations are associated with vitamin D-dependent rickets type 2A, interacted with 49 molecules with the highest degree, followed by *RUNX2* (47 molecules), *GJA1* (25 molecules), *SLC4A1* (23 molecules), and *ITGB4* (21 molecules) (Figure 4; Supplementary Table S9). Molecules that interacted with *VDR* were significantly enriched in the steroid hormone-mediated signaling pathway (adjusted *p*-value = 0) and in vitamin D receptor binding (adjusted *p*-value *=* 1.23 × 10^−9^).

We hypothesized that genes associated with amelogenesis imperfecta were enriched in some specific tissues with common biological processes. To identify the tissue specificity of amelogenesis imperfecta-associated genes, we explored their expression across 49 tissues profiled in the Genotype-Tissue Expression project [36]. The expression of amelogenesis imperfecta-associated genes was reported in various tissues, including the testes, artery, pituitary, thyroid, brain, adipose tissues, and lungs (Supplementary Figure S1a). Gene expression varied in the different tissues, but some genes, such as *GJA1*, *KLK4*, and *FAM20C*, were ubiquitously expressed in all tissues (Supplementary Figure S1b). With differing distribution and expression levels of these genes, tissues that show a large number of amelogenesis imperfecta-associated genes may be frequently affected in the syndromic form of the disease.

**Figure 5:**
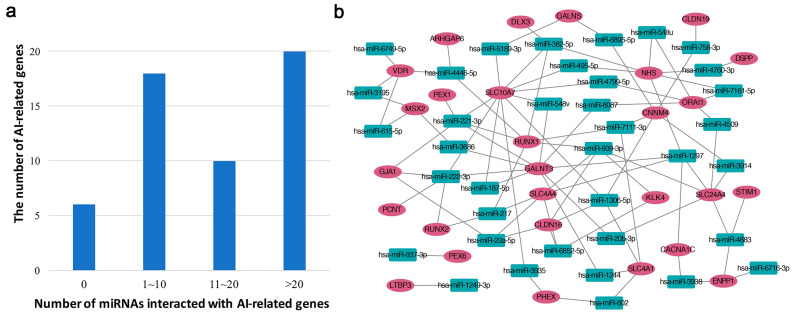
Characteristics of miRNAs associated with amelogenesis imperfecta (AI). (**a**) The distribution of miRNAs interacting with amelogenesis imperfecta-associated genes. (**b**) The enriched miRNA regulation network. Red circles denote genes related with amelogenesis imperfecta. Green squares denote human miRNAs enriched with amelogenesis imperfecta-related genes. An edge is laid when interaction between miRNA and gene has been reported in the database.

Next, to identify amelogenesis imperfecta-associated genes regulated by miRNAs, we performed bioinformatic analyses using multiple tools and databases. We found a total of 48 amelogenesis imperfecta-associated genes that were targeted by miRNAs. Among them, 20 genes were targeted by more than 20 miRNAs (Figure 5a), and four genes (*ACP4*, *COL17A1*, *FAM20C*, and *PEX1*) were targeted by only one miRNA, respectively (Supplementary Table S10). In addition, six genes (*AMBN*, *AMTN*, *FAM20A*, *GPR68*, *ODAPH*, and *SLC13A5*) have not yet been reported as associated with miRNAs. Finally, to identify miRNAs that regulate the expression of genes associated with amelogenesis imperfecta, we applied the Fisher’s exact test for miRNA target gene enrichment. We detected 37 miRNAs that were significantly (*p*-value < 0.05) enriched with amelogenesis imperfecta-associated genes (Table 4). To better represent the gene regulation of these miRNAs, we constructed a miRNA regulation network (Figure 5b).

### 2.3. Experimental Validation

**Figure 6:**
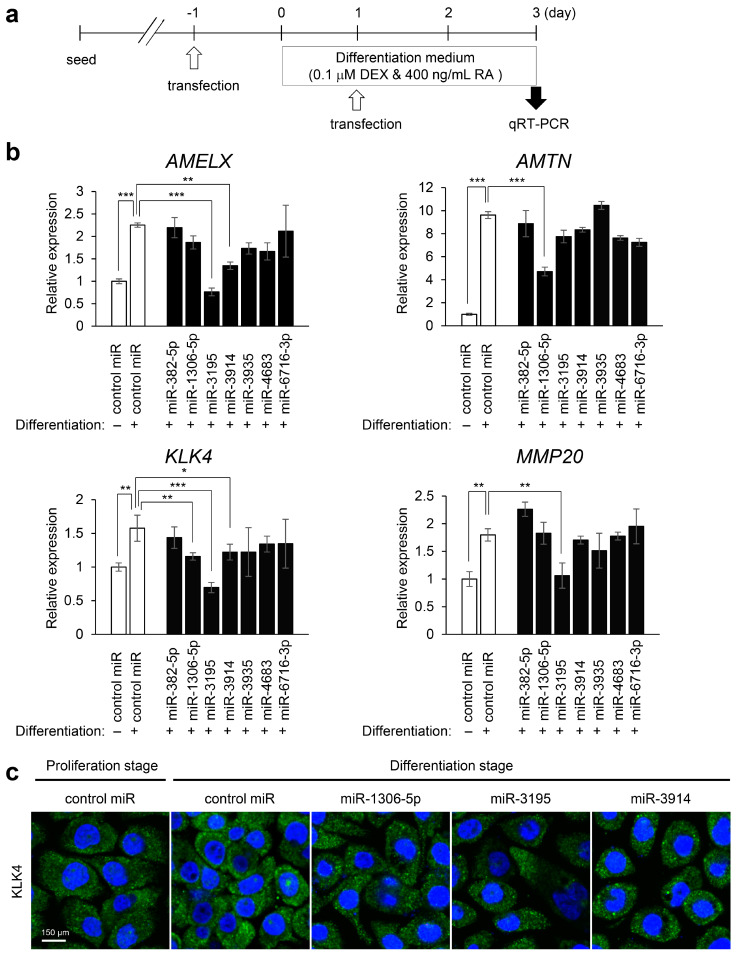
Effects of overexpression of candidate miRNAs on ameloblast differentiation. (**a**) Scheme of the experimental timeline. (**b**) Gene expression of AMELX, AMTN, KLK4, and MMP20 after treatment with a mimic of control or candidate miRNAs in AM-1 cells. * *p* < 0.05; ** *p* < 0.01, *** *p* < 0.001. (**c**) Immunocytochemistry analysis for KLK4 (green) in AM-1 cells under the indicated conditions. The nuclei were counterstained with 4’,6’-diamidino-2-phenylindole [DAPI (blue)]. Scale bar, 150 μm.

To validate the findings across the bioinformatic analyses, we performed ameloblast differentiation assays in AM-1 cells overexpressing the top seven candidate miRNAs (miR-3195, miR-382-5p, miR-1306-5p, miR-4683, miR-6716-3p, miR-3914, and miR-3935). To test whether AM-1 cells can differentiate into secretory and mature ameloblasts, which are determined by expression of genes related to amelogenesis such as the *AMELX*, *AMTN*, *KLK4*, and *MMP20* genes, we treated the cells with retinoic acid and dexamethasone, which is known to induce amelogenesis in mouse ameloblast-like cell lines [37,38,39]. We found that, under differentiation conditions with retinoic acid and dexamethasone, the expression of the genes related to ameloblast differentiation was significantly upregulated in AM-1 cells (Supplementary Figure S2). Among them, retinoic acid (0–400 μg/mL) with dexamethasone (0.1 µM) induced the expression of *AMELX*, *AMTN*, *KLK4*, and *MMP20* in a dose-dependent manner. The expression of *AMBN* and *ENAM* was also induced, but the upregulation was less than that for the others. In addition, the expression of *AMELX*, *KLK4*, and *MMP20* was not induced at differentiation Day 1 (Supplementary Figure S3a,b). Therefore, we used the expression of *AMELX*, *AMTN*, *KLK4*, and *MMP20* at Day 3 of the differentiation for the evaluation of the effects on ameloblast differentiation. We found that among seven candidate miRNAs, three miRNAs (miR-3195, miR-1306-5p, and miR-3914) could significantly suppress the expression of genes related to ameloblast differentiation (Figure 6). For instance, treatment with a miR-3195 mimic suppressed expression of *AMELX*, *KLK4*, and *MMP20*. Treatment with a miR-1306-5p mimic suppressed expression of *AMTN* and *KLK4*, and treatment with a miR-3914 mimic suppressed expression of *AMELX* and *KLK4*. Thus, overexpression of miR-3195, miR-1306-5p, and miR-3914 disturbed ameloblast differentiation in AM-1 cells, while treatment with a mimic for either miR-382-5p, miR-4683, miR-6716-3p, or miR-3935 failed to affect ameloblast differentiation in AM-1 cells (Figure 6b). We also analyzed the expression of miR-1306-5p and miR-3914, under cell proliferation and differentiation conditions, in AM-1 cells. We found that expression of miR-1306-5p and miR-3914 was low for both proliferation and differentiation conditions, suggesting that these miRNAs do not play a role in ameloblast differentiation (Supplementary Figure S4). We conducted immunocytochemical analyses for KLK4, which was induced under ameloblast differentiation conditions and suppressed with mimics for miR-1306-5p, miR-3195, and miR-3914, and confirmed that these miRNA mimics inhibited KLK4 expression under differentiation conditions (Figure 6c). Similarly, miR-3195 mimic inhibited MMP20 expression under differentiation conditions (Supplementary Figure S5). Thus, each miRNA mimic differently affected the expression of genes related to amelogenesis. One possibility is that these genes were direct downstream target genes for the miRNAs tested, while another possibility is that the sensitivity to each miRNA may differ. Since there are few candidate genes related to amelogenesis in the pool of miRNA prediction, we tested whether *AMELX*, *AMTN*, *KLK4*, and *MMP20* were direct targets of each miRNA at Day 1 of the differentiation and found that miR-1306-5p mimic, but not miR-3195 and miR-3914, downregulated *AMTN* expression compared to the control (Supplementary Figure S3b). In addition, miR-1306-5p inhibitor upregulated *AMTN* expression (Supplementary Figure S3f). These observations suggest that *AMTN* is a direct target of miR-1306-5p (Supplementary Figure S3).

**Figure 7:**
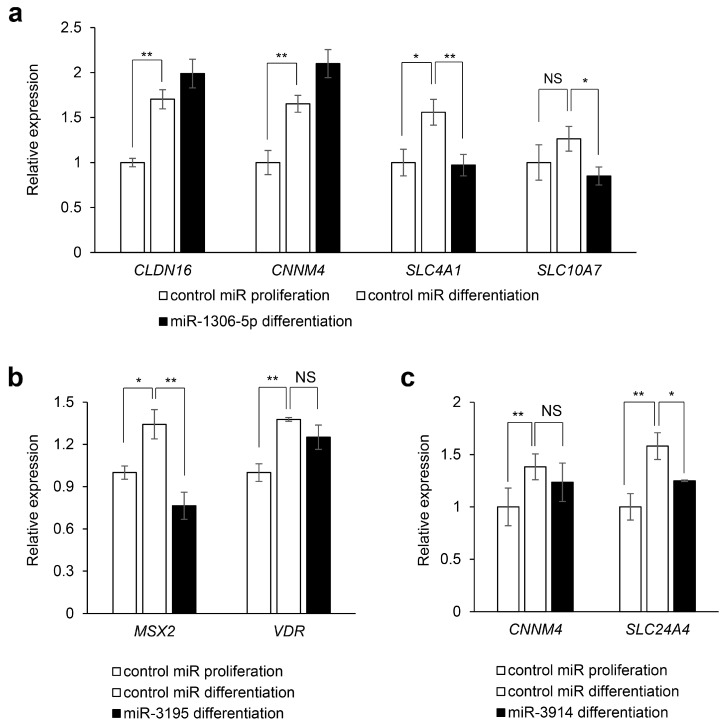
Effects of overexpression of candidate miRNAs on target gene expression. (**a**) Quantitative real-time polymerase chain reaction (RT-PCR) analyses for the target gene expression after treatment with control and miR-1306-5p mimic. * *p* < 0.05; ** *p* < 0.01; NS, not significant. Control miR proliferation: cells were treated with control miR mimic during the cell proliferation phase. Control miR differentiation: cells were treated with control miR mimic under differentiation conditions for 3 days. (**b**) Quantitative RT-PCR analyses for target gene expression after treatment with control and miR-3195 mimic. * *p* < 0.05; ** *p* < 0.01; NS, not significant. (**c**) Quantitative RT-PCR analyses for target gene expression after treatment with control and miR-3914 mimic. * *p* < 0.05; ** *p* < 0.01; NS, not significant.

Next, to investigate the miRNA–gene regulatory mechanisms, we analyzed the target gene expression predicted for each miRNA in AM-1 cells after treatment with each miRNA mimic (Figure 7). We validated that miR-1306-5p suppressed the expression of *SLC4A1* and *SLC10A7* (Figure 7a), and that miR-3195 downregulated the expression of *MSX2* (Figure 7b) and miR-3914 downregulated the expression of *SLC24A4* (Figure 7c). We also confirmed that expression of AMELX, KLK4, and MMP20 was not altered by overexpression of miR-1306-5p, miR-3195, and miR-3914 at Day 1 of ameloblast differentiation (Supplementary Figure S3b). We measured the expression of genes related to amelogenesis under differentiation conditions for 24 h and confirmed that the predicted downstream target genes were specifically downregulated following treatment with miRNA mimic (Supplementary Figure S3c–e). Taken together, our results demonstrate that the miR-1306-5p‒*SLC4A1*/*SLC10A7*, miR-3195‒*MSX2*, and miR-3914‒*SLC24A4* regulatory mechanisms are crucial for ameloblast differentiation.

## 3. Discussion

Ameloblasts are derived from the oral epithelium [15]. Therefore, mutations in *COL17A1*, *DLX3*, *GALNT3*, *GJA1*, *ITGB4*, *LAMA3*, *LAMB3*, and *TP63*, which are specifically expressed in epithelial cells, are responsible for amelogenesis imperfecta as well as other ectodermal defects. Several amelogenesis imperfecta-associated genes (*CLDN6*, *CLDN9*, *COL17A1*, *GJA1*, *ITGB4*, and *ITGB6*) are grouped into the cell adhesion molecules, which are important for ectodermal functions. By contrast, some amelogenesis imperfecta-associated genes (e.g., *PEX1*, *PEX6*, *ROGDI*, and *SLC13A5*) are expressed in mesenchymal tissues derived from cranial neural crest cells. This suggests that tissue–tissue interactions contribute to proper ameloblast differentiation and function and that dysregulation of these genes is associated with the pathogenesis of amelogenesis imperfecta.

Among the genes associated with the condition, three genes (*CACNA1C*, *KCNJ1*, and *ORAI1*) are grouped into ion channels, and seven genes (*CNNM4*, *SLC4A1*, *SLC4A4*, *SLC10A7*, *SLC13A5*, *SLC24A4*, and *STIM1*) are related to an ion transporter or ion exchanger and sensor. This indicates that calcium ion transportation and the movement of other ions are important for proper enamel development. In addition, 10 genes (*AMBN*, *AMELX*, *AMTN*, *DMP1*, *DSPP*, *ENAM*, *LAMA3*, *LAMB3*, *ODAPH*, and *LTBP3*) are grouped in “extracellular matrixes”, and 13 genes (*ACP4*, *CYP27B1*, *ENPP1*, *FAM20C*, *GALNS*, *GALNT3*, *GLA*, *KLK4*, *MMP20*, *PEX1*, *PEX6*, *PEX26*, and *PHEX*) are grouped in “enzymes”. Among them, FAM20C, KLK4, and MMP20 directly function to modify the enamel matrix. This indicates that mutations in genes related to extracellular matrix formation and degradation are also involved in the pathogenesis of amelogenesis imperfecta. As highlighted in the bioinformatic analyses, genes associated with amelogenesis imperfecta are involved in kidney and bone diseases, where these genetic mutations cause defects in the syndromic forms. For example, mutations in six of the amelogenesis imperfecta-associated genes (*CLDN16*, *CLDN19*, *FAM20A*, *KCNJ1*, *SLC4A1*, and *WDR72*) are associated with nephrocalcinosis, hypercalciuria, and renal failure. Seven of the amelogenesis imperfecta-associated genes (*CYP27B1*, *EPNN1*, *PHEX*, *SLC4A1*, *SLC4A4*, *VDR*, and *WDR72*) are known to cause rickets when mutated. In addition, mutations in *DLX3*, *FAM20C*, *LTBP3*, *PCNT*, *RUNX2*, and *SLC10A7* have been found in skeletal and bone dysplasias. Thus, the information on functional enrichment and gene expression will help identify and characterize the syndromes’ clinical features.

Through the systematic review and manual search, we identified 56 genes as ameloblast imperfecta-associated genes and predicted 37 miRNAs to be involved in amelogenesis imperfecta. In this study, to evaluate the miRNA–gene regulation in amelogenesis in humans, we used AM-1 cells [40], instead of the widely used mouse ameloblast cell line, LS-8 cells [41]. We found that overexpression of miR-1306-5p, miR-3195, and miR-3914 inhibits ameloblast differentiation. miR-1306 is upregulated in the plasma of mothers who are delivering babies with fatal growth restriction [42]. miR-3195 is expressed in several cancers and can induce apoptosis, acting as a tumor suppressor [43,44,45], whereas miR-3914′s expression profile has not been reported. In addition, we found that *AMTN* is a target of miR-1306-5p during amelogenesis. Since there are few genes in the miRTarBase database, our study provides a new map of miRNA–gene interactions during amelogenesis. Environmental factors, such as excessive fluoride, nutritional deficiency, trauma, chemical therapy, ingestion of chemicals, and infection, can cause enamel defects (Supplementary Table S3.6) and influence miRNA expression [46,47,48]. Some of these conditions may alter miRNA expression, which can cause enamel defects by suppressing genes that are important for amelogenesis.

## 4. Materials and Methods

### 4.1. Systematic Review

A literature search was conducted, following the published guidelines set forth by the Preferred Reporting Items for Systematic Reviews and Meta-Analyses (PRISMA), using the three main scientific literature databases: Scopus (Elsevier, Inc. Frisco, CO, USA), PubMed (NLM), and Embase (Ovid). Terms for the search included amelogenesis imperfecta, genetics (gene mutations), and humans. A combination of Medical Subject Headings (MeSH) terms and titles, abstracts, and keywords were used to formulate the initial PubMed search string, and then adapted to search the other databases. The articles meeting the following eligibility criteria were included in the systematic review: described genes causing or potentially associated with amelogenesis imperfecta in humans, were published as original articles, including case reports (excluding review articles, editorials, dissertations, conference proceedings, comments, or books), and were written in English. After screening for articles using the criteria above, the following articles were manually excluded: amelogenesis imperfecta was not described, only treatments, follow-up, diagnostic, and public health/prevalence of amelogenesis imperfecta were described, gene mutations associated with amelogenesis imperfecta in humans were not described, and the articles failed to fit in any of the above exclusion criteria but did not have amelogenesis imperfecta candidate genes or related information. All the citations found in the search process were stored in RefWorks (ProQuest), and duplicates were excluded. The search strategies and results were tracked using the Rayyan software designed for systematic reviews (https://rayyan.qcri.org/users/sign_in (accessed on 15 January 2021)). To check the reliability of study selection between screeners, the Cohen’s Kappa test was applied, using randomly selected samples of 216 articles screened by titles and abstracts. After achieving a >90% score for the Cohen’s Kappa test, all the titles and abstracts of the articles found through the database search were independently examined. The full text of articles not excluded in the above process was manually reviewed, and all results from the screening were recorded in the Primary Excel Workbook.

### 4.2. Bioinformatic Analyses

A hypergeometric test and functional enrichment analysis of the genes related to amelogenesis imperfecta were performed using WebGestalt (2019 version) [49]. To report reliable results, the pathways and gene ontology (GO) terms with an adjusted *p*-value < 0.05 were selected (adjusted by Benjamini-Hochberg Procedure, false discovery rate (FDR) [50]). Furthermore, the number of genes was set at 5 to 200 to avoid too many GO terms, redundant terms, or insufficient statistical power. To better interpret the results, an amelogenesis imperfecta-related pathway network was constructed using the plug-in module ClueGO (v. 2.5.7) [37] in Cytoscape (v. 3.6.0) [38]. In this network, a node represents a gene or a term, while an edge indicates a gene that belongs to a term. The protein–protein interactions (PPIs) between amelogenesis imperfecta-related molecules were analyzed in five databases: Human Protein Reference Database (HPRD) [31], BioGrid [32], IntAct [33], Molecular INTeraction database (MINT) [34], and Database of Interacting Proteins ( DIP) [35]. A tissue-specific enrichment analysis was conducted using an R-package deTS [39], which is established using the GTEx database V7 [36] collected from 53 non-disease (normal) tissue sites across nearly 1000 individuals. The candidate miRNA–gene pairs were identified using bioinformatic tools with multiple target prediction algorithms, including TargetScan (v. 7.1) [51], miRanda (August 2010 Release) [52], Perforation Inflow Test Analysis (PITA) (version 6) [53], and the miRTarBase (Release 7.0), which comprises experimentally validated miRNA–gene interactions.

### 4.3. Cell Culture

Human ameloblastoma AM-1 cells were cultured in keratinocyte-serum free medium (SFM) supplemented with 50 μg/mL bovine pituitary extract (BPE, 17005042, ThermoFisher, Waltham, MA, USA) and 1% penicillin-streptomycin at 37 °C with 5% CO_2_, as previously described [40]. Cells were plated onto cell culture plates at a density of 50,000 cells/mL and cultured until they reached 80% confluency. The cells were then treated with mimic for miR-3195, miR-382-5p, miR-1306-5p, miR-4683, miR-6716-3p, miR-3914, miR-3935, or control (mirVana miRNA mimic, ThermoFisher Scientific, Waltham, MA, USA), using Lipofectamine RNAiMAX transfection reagent (ThermoFisher Scientific) according to the manufacturer’s protocol (12 pmol of mimic with 1.2 µL of transfection reagent in 500 µL of keratinocyte-SFM medium). After 24 h, the differentiation medium [400 ng/mL retinoic acid (R2625, Sigma Aldrich, St. Louis, MO, USA) and 0.1 μM dexamethasone (D4902, Sigma Aldrich) in keratinocyte-SFM with BPE] was replaced and the cells were cultured for three days.

### 4.4. Quantitative Revers Transcription Polymerase Chain Reaction (RT-PCR)

Total RNA isolated from AM-1 cells (*n* = 6 per treatment group) was extracted using the QIAshredder and RNeasy mini extraction kit (QIAGEN, Hilden, Germany), as previously described [54]. Briefly, 1 µg of total RNA was reverse-transcribed to cDNA with the iScript Reverse Transcription Super Mix (BioRad, Hercules, CA, USA), and the resulting cDNA was amplified with the iTaq Universal SYBER Green Super Mix (BioRad) using a CFX96 Touch Real-Time PCR Detection System (BioRad). The expression of mRNA was normalized by the glyceraldehyde 3-phosphate dehydrogenase gene (*GAPDH*). The following PCR primers were used for further specific analysis: *AMELX*, 5′-TGCCTCTACCACCTCATCCT-3′ and 5′-TGGAGTCATGGAGTGTTGGC-3′; *AMTN*, 5′-GTACCCAGACCCACCCATTG-3′ and 5′-CATCTGTGCCACTGGGAGTT-3′; *CLDN16*, 5′-TGACTCTCTGGAGGTGAGCA-3′ and 5′-AGGGATGCTCCGCAAGTATG-3′; *CNNM4*, 5′-GAGCTGCAACAAGTCGTGTG-3′ and 5′-CAGTGAGTCCTTGTCCGTCC-3′; *KLK4*, 5′-CCGCACACTGTTTCCAGAAC-3′ and 5′-CGAAGCAATGCTGATGCTCC-3′; *MMP20*, 5′-GGAGGAACAACTACCGCCTC-3′ and 5′-GGCCAAAGAACGCTTGTAGC-3′; *MSX2*, 5′-AATGACTTGTTTTCGCCCGAC-3′ and 5′-CATATGTCCTCCTACTCCTGCC-3′; *SLC4A1*, 5′-GGAATCAGTGGACTCCGAGG-3′ and 5′-AAATGAGGGGCCTGAAGTTGT-3′; *SLC10A7*, 5′-CGTCCATAGGGGTGAATGGG-3′ and 5′-AATATTGCAGCTGCCTCATTTCC-3′; *SLC24A4*, 5′-CAGGAGGCGAGAGATGCTG-3′ and 5′- CAGAAGCTGTTTTGTGCCCC-3′; *VDR*, 5′-CTGACTAGGACAGCCTGTGG-3′ and 5′- CGCAGGAAAGGGGTTAGGTT-3′; *GAPDH*, 5′- GACAGTCAGCCGCATCTTCT-3′ and 5′- GCGCCCAATACGACCAAATC-3′. miRNA extraction was performed with the QIAshredder and miRNeasy Mini Kit (QIAGEN), according to the manufacturer’s instructions. miR expression was measured with the Taqman Fast Advanced Master Mix and Taqman Advanced miR cDNA Synthesis Kit (Thermo Fisher Scientific). Probes for miR-1306-5p (478701_mir), miR-3914 (479736_mir), and miR-26a-5p (477995_mir) were purchased from Thermo Fisher Scientific. The amount of each miRNA was normalized by miR-26a-5p.

### 4.5. Immunofluorescence Analysis

The cells were plated onto ibiTreat 8-well μ-slides (ibidi GmbH, Munich district, Germany) at a density of 5000/chamber and cultured until 80% confluency. Then, cells were treated with mimic for miR-3195, miR-1306-5p, miR-3914, or control using Lipofectamine RNAiMAX transfection reagent (4.8 pmol of mimic with 0.48 µL of transfection reagent in 200 µL of keratinocyte-SFM medium). After 24 h, the cells were cultured with differentiation medium for 72 h. Immunofluorescence analysis was performed, as previously described [55], using rabbit polyclonal antibodies against KLK4 (PA5-109888, Thermo Fisher Scientific, 1:200) and MMP20 (55467-1-AP, Proteintech, 1:250). Images were taken with a confocal microscope (Ti-E, Nikon USA, Melville, NY, USA).

### 4.6. Statistical Analysis

The two-tailed Student’s *t*-test or post hoc Tukey–Kramer test was applied for the statistical analysis. A *p*-value < 0.05 was considered statistically significant. For all graphs, data are represented as mean ± standard deviation (SD).

## Figures and Tables

**Table 1:** Genes associated with isolated or syndromic amelogenesis imperfecta.

Isolated vs. Syndromic	Genes
Isolated	*ACP4, AMBN, AMELX, AMTN, ARHGAP6, DLX3, ENAM, FAM83H, GPR68, ITGB6, KLK4, LAMA3, LAMB3, MMP20, ODAPH, SLC24A4, WDR72*
Syndromic	*AIRE, ALPL, AMELX, CACNA1C, CLDN16, CLDN19, CNNM4, COL17A1, CYP27B1, DLX3, DMP1, DSPP, ENPP1, FMA20A, FAM20C, GALNS, GALNT3, GJA1, GLA, ITGB4, KCNJ1, LAMA3, LAMB3, LTBP3, MSX2, NHS, ORAI1, PCNT, PEX1, PEX6, PEX26, PHEX, RELT, ROGDI, RUNX1, RUNX2, SLC4A1, SLC4A4, SLC10A7, SLC13A5, STIM1, TP63, VDR, WDR72*

Underlined: categorized in both isolated and syndromic amelogenesis imperfecta.

**Table 2:** Genes per inheritance type.

Inheritance Type	Genes
autosomal recessive	*ACP4*, *AIRE*, *ALPL*, *AMBN*, *CLDN16*, *CLDN19*, *CNNM4*, *CYP27B1*, *DMP1*, *ENPP1*, *FAM20A*, *FAM20C*, *GALNS*, *GALNT3*, *GPR68*, *ITGB4*, *ITGB6*, *KCNJ1*, *KLK4*, *LAMA3*, *LAMB3*, *LTBP3*, *MMP20*, *ODAPH*, *ORAI1*, *PCNT*, *PEX1*, *PEX6*, *PEX26*, *RELT*, *ROGDI*, *SLC4A1*, *SLC4A4*, *SLC10A7*, *SLC13A5*, *SLC24A4*, *STIM1*, *TP63*, *VDR*, *WDR72*
autosomal dominant	*ALPL, AMBN*, *AMTN*, *CACNA1C*, *CNNM4*, *COL17A1*, *DLX3*, *DSPP*, *ENAM*, *FAM83H*, *GJA1*, *LAMA3*, *LAMB3*, *MSX2*, *RUNX2*, *SLC4A1*
X-linked recessive	*ARHGAP6*
X-linked dominant	*AMELX*, *GLA*, *NHS*, *PHEX*

Underlined: categorized in both autosomal recessive and dominant type.

**Table 3:** Genes grouped by amelogenesis imperfecta phenotype.

Amelogenesis Imperfecta Category	Genes
hypoplastic	*ACP4, AIRA, ALPL, AMBN, AMELX, CLDN16, CLDN19, CNNM4, COL17A1, CYP27B1, DLX3, DSPP, ENPP1, ENAM, FAM20A, FAM20C, GALNT3, GJA1, GLA, ITGB4, ITGB6, KCNJ1, LAMA3, LAMB3, LTBP3, MMP20, ODAPH, PEX1, PEX6, PEX26, PHEX, ROGDI, RUNX1, RUNX2, SLC4A4, SLC10A7, SLC13A5, TP63, VDR, WDR72*
hypomaturation	*AMELX, CLDN16, CLDN19, CNNM4, KLK4, MMP20, MSX2, ROGDI, RUNX2, SLC24A4, STIM1, TP63, WDR72*
hypomineralized/hypocalcified	*AMBN, AMELX, AMTN, CNNM4, DMP1, DSPP, ENAM, FAM83H, GPR68, ITGB6, KLK4, MMP20, ODAPH, ORAI1, PEX1, RELT, ROGDI, SLC10A7, SLC24A4, STIM1, TP63, WDR72*
hypoplastic/hypomature enamel with taurodontism	*ALPL, DLX3, LAMB3*
not specified	*ARHGAP6, CACNA1C, GALNS, NHS, PCTN, SLC4A1*

**Table 4:** MicroRNA (miRNA) enrichment analysis of amelogenesis imperfecta-associated genes.

miRNAs	Gene Symbol	# Target	# Overlap	*p*-Value
hsa-miR-3195	*MSX2, VDR*	23	2	0.00287231
hsa-miR-382-5p	*DLX3, SLC10A7, NHS, RUNX1*	186	4	0.003965
hsa-miR-1306-5p	*SLC4A1, CNNM4, SLC10A7, CLDN16*	199	4	0.00503641
hsa-miR-4683	*SLC24A4, ENPP1, STIM1*	108	3	0.00629218
hsa-miR-6716-3p	*ENPP1*	4	1	0.01385705
hsa-miR-3914	*SLC24A4, CNNM4*	56	2	0.01624945
hsa-miR-3935	*PHEX, SLC10A7*	56	2	0.01624945
hsa-miR-23a-5p	*SLC4A4, GJA1, CLDN16*	155	3	0.0167345
hsa-miR-4509	*SLC24A4, ORAI1*	57	2	0.01680318
hsa-miR-1244	*GALNT3, SLC4A1*	59	2	0.01793454
hsa-miR-939-3p	*KLK4, RUNX1, SLC4A4, SLC24A4, CLDN16*	439	5	0.01799166
hsa-miR-3938	*ENPP1, CACNA1C*	65	2	0.02151565
hsa-miR-802	*PHEX, SLC4A1*	68	2	0.0234085
hsa-miR-615-5p	*MSX2, VDR*	70	2	0.02470722
hsa-miR-4760-3p	*DSPP, NHS*	71	2	0.02536746
hsa-miR-221-3p	*GALNT3, GJA1, PEX1, RUNX1, SLC10A7*	489	5	0.02716289
hsa-miR-1297	*GALNT3, CACNA1C, SLC4A4, SLC24A4, NHS*	496	5	0.02864777
hsa-miR-6895-5p	*GALNS, CNNM4*	83	2	0.03383429
hsa-miR-1249-3p	*LTBP3*	10	1	0.03428995
hsa-miR-3686	*GALNT3, MSX2*	84	2	0.03458354
hsa-miR-187-5p	*GALNT3, SLC10A7*	84	2	0.03458354
hsa-miR-222-3p	*GALNT3, PCNT, GJA1, RUNX2, SLC10A7*	523	5	0.03485762
hsa-miR-937-3p	*PEX6*	11	1	0.03765479
hsa-miR-217	*SLC4A4, RUNX2, RUNX1*	219	3	0.04067934
hsa-miR-7111-3p	*SLC4A1, CNNM4, RUNX1*	219	3	0.04067934
hsa-miR-495-5p	*NHS, SLC10A7*	92	2	0.04080612
hsa-miR-6749-5p	*VDR*	12	1	0.04100813
hsa-miR-5189-3p	*GALNS, SLC10A7*	94	2	0.04242339
hsa-miR-8087	*GALNT3, ORAI1*	94	2	0.04242339
hsa-miR-4799-5p	*ORAI1, SLC10A7*	94	2	0.04242339
hsa-miR-20b-3p	*GALNT3, SLC24A4*	95	2	0.04324099
hsa-miR-6852-5p	*SLC4A4, KLK4, CLDN16*	228	3	0.04494714
hsa-miR-4446-5p	*ARHGAP6, RUNX1, VDR*	231	3	0.04641776
hsa-miR-548u	*ORAI1, NHS*	101	2	0.04826897
hsa-miR-758-3p	*CNNM4, CLDN19*	102	2	0.04912692
hsa-miR-7161-5p	*ORAI1, NHS*	103	2	0.04999043
hsa-miR-548v	*GALNT3, SLC10A7*	103	2	0.04999043

## Data Availability

The datasets generated during and/or analyzed during the current study are available from the corresponding author upon reasonable request.

## Supplementary Materials

The following are available online at https://www.mdpi.com/1422-0067/22/4/2202/s1, Supplementary Figure S1: Tissue-specific expression of amelogenesis imperfecta-associated genes using GTEx data. Supplementary Figure S2: Ameloblast differentiation in AM-1 cells. Supplementary Figure S3: miRNA expression in AM-1 cells. Supplementary Figure S4: Effects of miR-3195 overexpression on MMP20 expression in AM-1 cells. Supplementary Figure S5: Effects of overexpression of candidate miRNAs on ameloblast differentiation and predicted target genes. Supplementary Table S1: microRNAs related to enamel formation. Supplementary Table S2: Amelogenesis imperfecta candidate genes in humans. Supplementary Table S3.1: Amelogenesis imperfecta candidate genes identified through systematic review. Supplementary Table S3.2: Amelogenesis imperfecta candidate genes identified through manual search. Supplementary Table S3.3: Syndromes with enamel defects identified through systematic review. Supplementary Table S3.4: Syndromes with enamel defects identified through manual search. Supplementary Table S3.5: Disease reported with enamel hypoplasia. Supplementary Table S3.6: Environmental factors reported with enamel defects. Supplementary Table S4: Number of patients, families, and reports for genes associated with amelogenesis imperfect. Supplementary Tabel S5: Syndromic amelogenesis imperfecta (AI) and enamel hypoplasia. Supplementary Tabel S6: Other dental phenotypes in individuals with mutations in genes associated with amelogenesis imperfect. Supplementary Table S7: Top20 enriched disease caused by amelogenesis imperfecta-associated genes. Supplementary Table S8: Functional enrichment analysis of amelogenesis imperfecta-associated genes. Supplementary Table S9: Molecules highlighted in bioinformatic analysis for protein-protein interactions. Supplementary Table S10: Predicted microRNAs associated with ameogenesis imperfecta associated genes.
