# Supplementary material for: Overexpression of miR-1306-5p, miR-3195, and miR-3914 Inhibits Ameloblast Differentiation through Suppression of Genes Associated with Human Amelogenesis Imperfecta
Source: Int J Mol Sci. 2021 Feb 23;22(4):2202. doi: 10.3390/ijms22042202 (PMC7926528; doi:10.3390/ijms22042202)
Supplement: Supplementary file 1 [file ijms-22-02202-s001.zip › Human AI systematic review_Supplemental Figures S1-S5.pdf]

Figure S1

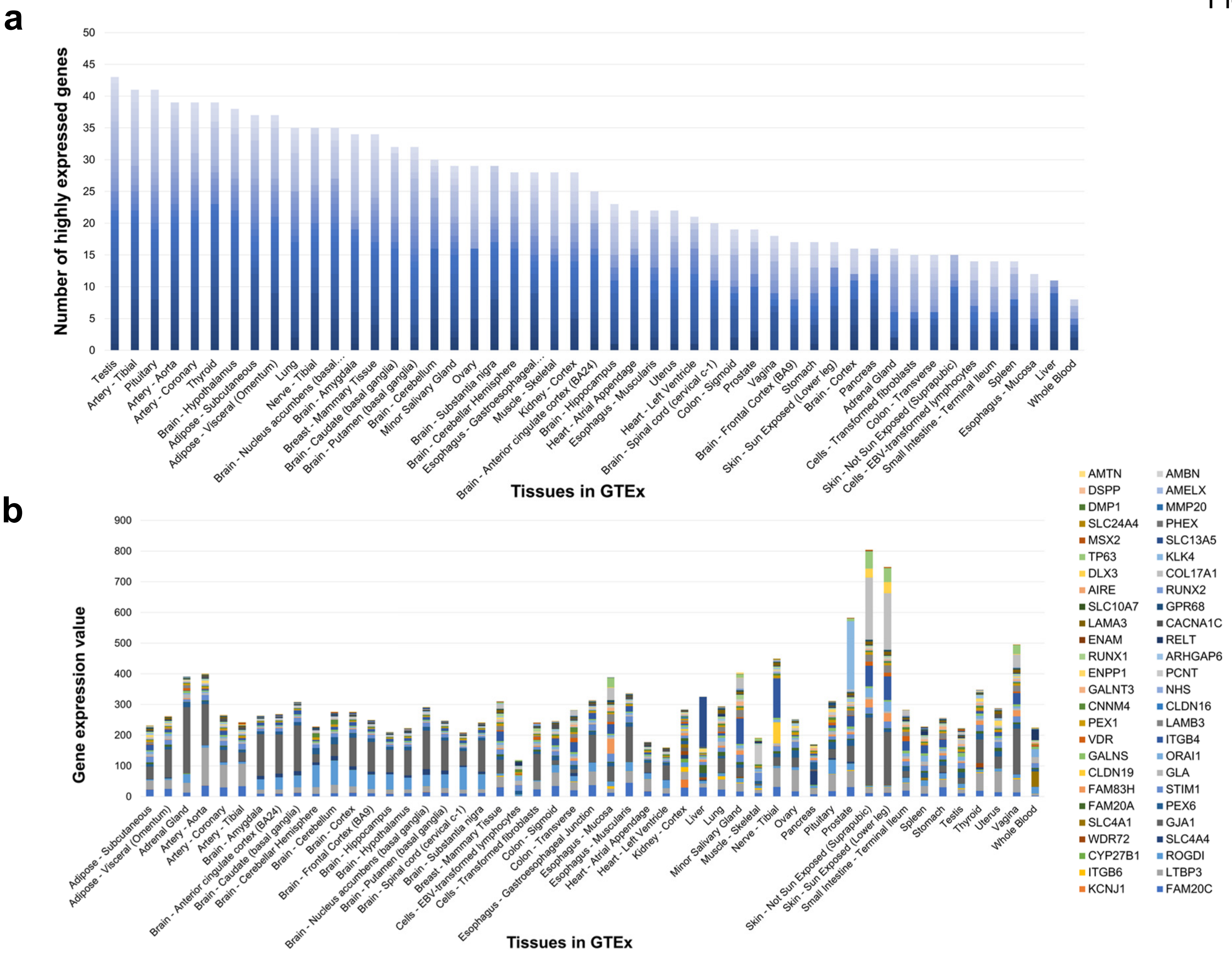

Figure S1. Tissue-specific expression of amelogenesis imperfecta-associated genes using GTEx data. (a) The number of amelogenesis imperfecta-associated genes that are highly expressed in the indicated tissues. (b) The gene expression level of the amelogenesis imperfecta-associated genes in the indicated tissues.

Figure S2

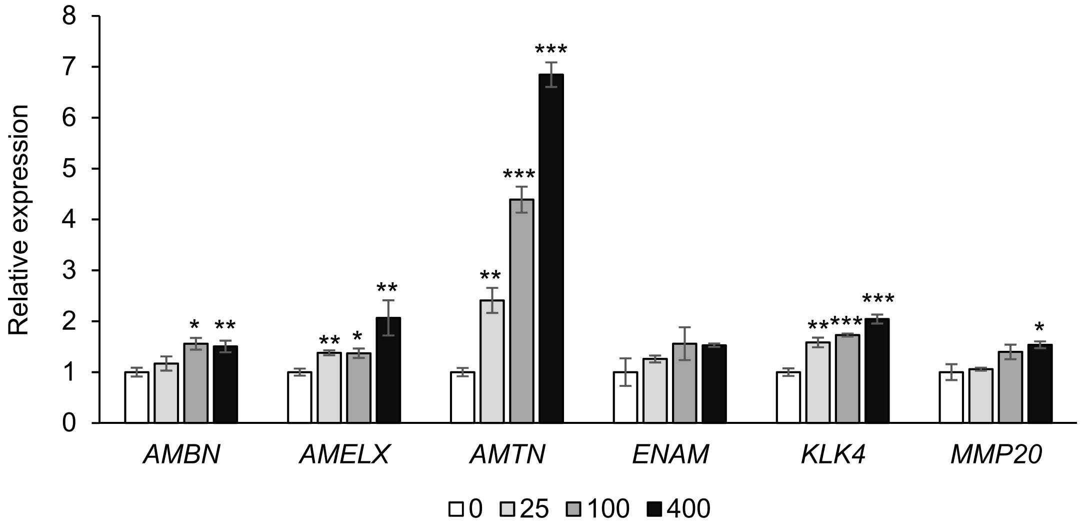

Figure S2. Ameloblast differentiation in AM-1 cells. Expressions of the indicated genes were analyzed 3 days after ameloblast differentiation induced with 0, 25, 100, or 400 ng/mL retinoic acid and 0.1 μM dexamethasone. *p < 0.05, **p < 0.01, ***p < 0.001 compared to the group treated with 0 μg/mL retinoic acid.

Figure S3

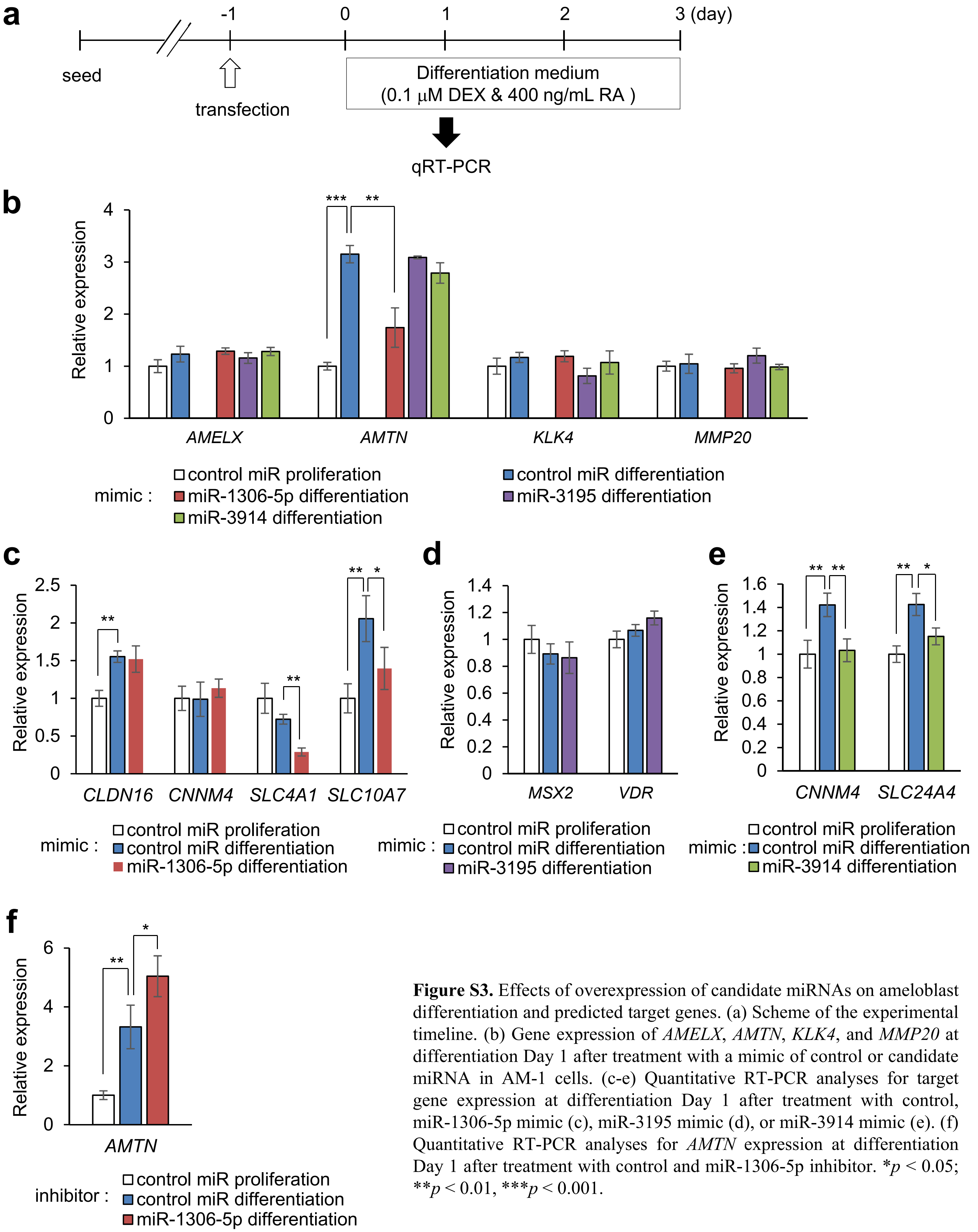

Figure S3. Effects of overexpression of candidate miRNAs on ameloblast differentiation and predicted target genes. (a) Scheme of the experimental timeline. (b) Gene expression of *AMELX*, *AMTN*, *KLK4*, and *MMP20* at differentiation Day 1 after treatment with a mimic of control or candidate miRNA in AM-1 cells. (c-e) Quantitative RT-PCR analyses for target gene expression at differentiation Day 1 after treatment with control, miR-1306-5p mimic (c), miR-3195 mimic (d), or miR-3914 mimic (e). (f) Quantitative RT-PCR analyses for *AMTN* expression at differentiation Day 1 after treatment with control and miR-1306-5p inhibitor. $*p < 0.05$; $**p < 0.01$, $***p < 0.001$.

a

	control mimic	miR-1306-5p mimic	miR-3914 mimic
miR-1306	1 ± 0.16	599040 ± 43107***	0.97 ± 0.065
miR-3914	1 ± 0.085	0.94 ± 0.30	454833 ± 18021***

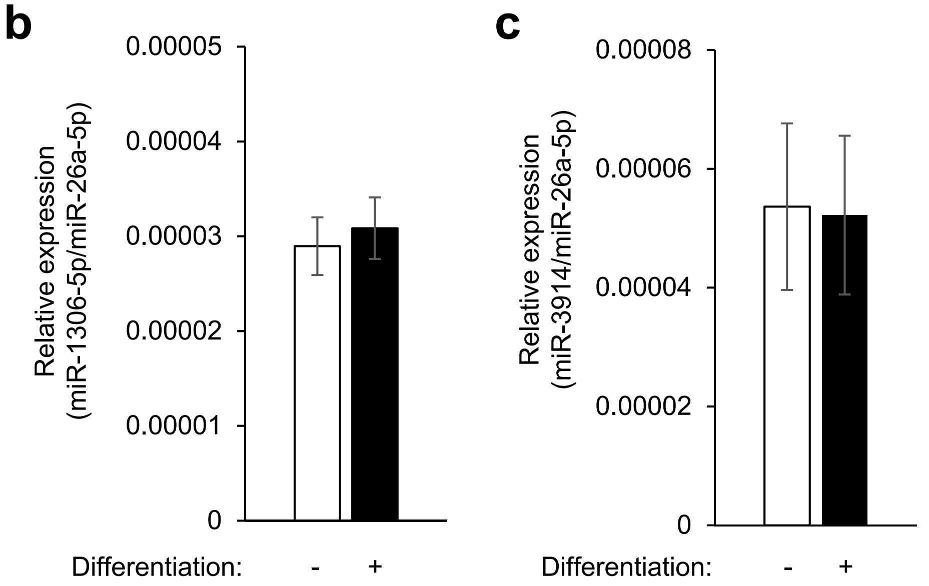

Figure S4. miRNA expression in AM-1 cells. (a) Expressions of miR-1306-5p and miR-3914 were analyzed under treatment with control, miR-1306-5p, or miR-3914 mimic for 24 hours. (b-c) Expressions of miR-1306-5p (b) and miR-3914 (c) were analyzed 3 days after ameloblast differentiation. ***$p < 0.001$ vs control mimic.

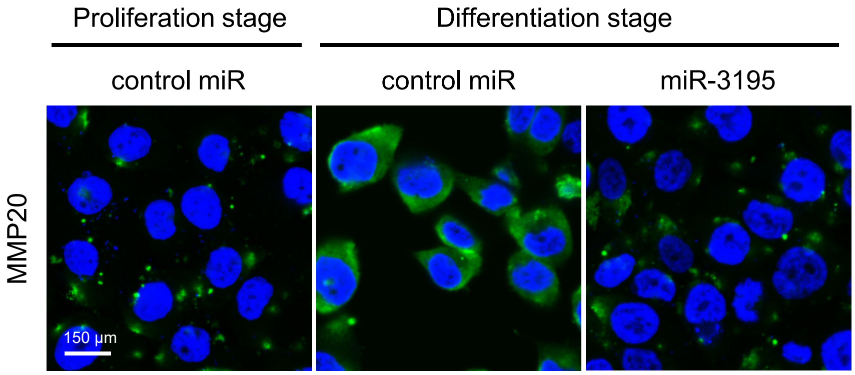

Figure S5. Effects of miR-3195 overexpression on MMP20 expression in AM-1 cells. Immunocytochemical analysis for MMP20 (green) in AM-1 cells after 3 days under differentiation conditions. The nuclei were counterstained with DAPI (blue). Scale bar, 150 μm.
