# Supplementary material for: Overexpression of miR-1306-5p, miR-3195, and miR-3914 Inhibits Ameloblast Differentiation through Suppression of Genes Associated with Human Amelogenesis Imperfecta
Source: Int J Mol Sci. 2021 Feb 23;22(4):2202. doi: 10.3390/ijms22042202 (PMC7926528; doi:10.3390/ijms22042202)
Supplement: Supplementary file 1 [file ijms-22-02202-s001.zip › Supplementary Table S2_Final_021221.docx]

**Supplementary Table S2.** Amelogenesis imperfecta candidate genes in humans

| **No** | **Gene symbol** | **Gene name** | **chromosome** | **syndromic/non-syndromic** | **AI type** |
| --- | --- | --- | --- | --- | --- |
| 1 | *ACP4 (aka ACPT)* | acid phosphatase 4 | 19q13.33 | Isolated | autosomal-recessive hypoplastic |
| 2 | *AIRE* | autoimmune regulator | 21q22.3 | Autoimmune polyendocrinopathy-candidiasis-ectodermal-dystrophy (APECED) | autosomal-recessive hypoplastic |
| 3 | *ALPL* | alkaline phosphatase, biomineralization associated | 1p36.12 | Hypophosphatasia | autosomal-recessive hypoplastic, autosomal-dominant hypoplastic, autosomal-recessive hypoplastic with taurodontism |
| 4 | *AMBN* | ameloblastin | 4q13.3 | Isolated | autosomal-dominant localized hypoplastic (AIH2), autosomal-dominant generalized hypoplastic, autosomal-recessive hypoplastic |
| 5 | *AMELX* | amelogenin X-linked | Xp22.2 | Kidney disease, Isolated | X-linked hypoplastic and/or hypomineralized  X-linked hypomaturation |
| 6 | *AMTN* | amelotin | 4q13.3 | Isolated (affected males have a more severe phenotype) | autosomal-dominant hypomineralized |
| 7 | *ARHGAP6* (Only when *AMELX* (exon 1 of *ARHGAP6*) is deleted) | Rho GTPase activating protein 6 | Xp22.2 | Isolated | X-linked-recessive hypoplastic |
| 8 | *CACNA1C* | calcium voltage-gated channel subunit alpha1 C | 12p13.33 | Timothy syndrome (TS) | poor enamel formation (no detailed information is available) |
| 9 | *CLDN16* (aka *PCLN1*) | claudin 16 | 3q28 | Familial hypomagnesaemia and hypercalciuria with nephrocalcinosis (FHHNC) | autosomal-recessive hypoplastic or hypomaturation |
| 10 | *CLDN19* | claudin 19 | 1p34.2 | Familial hypomagnesaemia and hypercalciuria with nephrocalcinosis (FHHNC) | autosomal-recessive hypoplastic or hypomaturation |
| 11 | *CNNM4* | cyclin and CBS domain divalent metal cation transport mediator 4 | 2q11.2 | Jalili syndrome | autosomal-recessive hypoplastic, autosomal-recessive-hypoplastic-hypomineralized, autosomal-recessive-hypomaturation/hypomineralized, autosomal-dominant hypoplastic |
| 12 | *COL17A1* | collagen type XVII alpha 1 chain | 10q25.1 | Junctional epidermolysis bullosa (JEB) | autosomal-dominant hypoplastic |
| 13 | *CYP27B1* | cytochrome P450 family 27 subfamily B member 1 | 12q14.1 | Vitamin D-dependent rickets type I (VDDRI) | autosomal-recessive hypoplastic |
| 14 | *DLX3* | distal-less homeobox 3 | 17q21.33 | Tricho-dento-osseous syndrome (TDO), Isolated | autosomal-dominant hypomaturation and hypoplastic, autosomal-dominant hypomaturation and hypoplasia with taurodontism (AIHHT) |
| 15 | *DMP1* | dentin matrix acid phosphoprotein 1 | 4q22.1 | Autosomal recessive form of hypophosphataemia (ARHP) | autosomal-recessive hypomineralized |
| 16 | *DSPP* | dentin sialophosphoprotein | 4q22.1 | Dentinogenesis imperfecta type II (DGI-II) | autosomal-dominant hypoplastic, autosomal-dominant hypocalcified |
| 17 | *ENPP1* | ectonucleotide pyrophosphatase/phosphodiesterase 1 | 6q23.2 | Autosomal recessive form of hypophosphataemic rickets (ARHR) | autosomal-recessive hypoplastic |
| 18 | *ENAM* | enamelin | 4q13.3 | Isolated | autosomal-dominant smooth or local hypoplastic,  autosomal-dominant hypocalcified, autosomal-recessive hypoplastic |
| 19 | *FAM20A* | FAM20A golgi associated secretory pathway pseudokinase | 17q24.2 | Enamel-renal syndrome (ERS), Enamel-renal-gingival syndrome (ERGS), Amelogenesis imperfecta-gingival fibromatosis (AIGFS) | autosomal-recessive hypoplastic |
| 20 | *FAM20C* | FAM20C golgi associated secretory pathway kinase | 7p22.3 | Non-lethal Raine syndrome, Familial hypophosphataemia | autosomal-recessive hypoplastic |
| 21 | *FAM83H* | family with sequence similarity 83 member H | 8q24.3 | Isolated | autosomal-dominant hypocalcification (ADHCAI) |
| 22 | *GALNS* | galactosamine (N-acetyl)-6-sulfatase | 16q24.3 | Morquio's syndrome (mucopolysaccharidosis type IVA) | autosomal-recessive dental defect |
| 23 | *GALNT3* | polypeptide N-acetylgalactosaminyltransferase 3 | 2q24.3 | Hyperphosphataemic familial tumoral calcinosis (HFTC) | autosomal-recessive hypoplastic |
| 24 | *GJA1* (aka *CX43*) | gap junction protein alpha 1 | 6q22.31 | Oculodentodigital dysplasia (ODDD) | autosomal-dominant hypoplastic |
| 25 | *GLA* | galactosidase alpha | Xq22.1 | Anderson-Fabry disease/ angiokeratoma corporis diffusum (ACD) | X-linked hypoplastic |
| 26 | *GPR68* | G protein-coupled receptor 68 | 14q32.11 | Isolated | autosomal-recessive hypomineralized |
| 27 | *ITGB4* | integrin subunit beta 4 | 17q25.1 | Epidermolysis bullosa with pyloric atresia (EB-PA) | autosomal-recessive hypoplastic |
| 28 | *ITGB6* | integrin subunit beta 6 | 2q24.2 | Isolated | autosomal-recessive pitted hypomineralized, pitted hypoplastic with hypomineralized |
| 29 | *KCNJ1* | potassium voltage-gated channel subfamily J member 1 | 11q24.3 | Bartter syndrome (BS) | autosomal-recessive hypoplastic |
| 30 | *KLK4* | kallikrein related peptidase 4 | 19q13.41 | Isolated | autosomal-recessive hypomaturation/hypomineralized |
| 31 | *LAMA3* | laminin subunit alpha 3 | 18q11.2 | Junctional epidermolysis bullosa (JEB)-generalized intermediate (non-Herlitz JEB), Laryngo-onycho-cutaneous (LOC) syndrome, Isolated | autosomal-recessive hypoplastic, autosomal-dominant pitted enamel |
| 32 | *LAMB3* | laminin subunit beta 3 | 1q32.2 | Junctional epidermolysis bullosa (JEB), Isolated | autosomal-recessive hypoplastic, autosomal-dominant hypoplastic, autosomal-dominant hypoplastic with taurodontism |
| 33 | *LTBP3* | latent transforming growth factor beta binding protein 3 | 11q13.1 | Brachyolmia with amelogenesis imperfecta, thoracic aortic aneurysms and dissections | autosomal-recessive hypoplastic |
| 34 | *MMP20* | matrix metallopeptidase 20 | 11q22.2 | Isolated | autosomal-recessive hypomaturation |
| 35 | *MSX2* | msh homeobox 2 | 5q35.2 | Amelogenesis imperfecta, cleft lip and palate, and polycystic kidney disease | autosomal-dominant hypomaturation |
| 36 | *NHS* | NHS actin remodeling regulator | Xp22.2-p22.13 | Nance-Horan syndrome (NHS) | X-linked hypoplastic, and screwdriver-shaped incisors, and bud molars |
| 37 | *ODAPH* (aka *C4orf26*) | odontogenesis associated phosphoprotein | 4q21.1 | Isolated | autosomal-recessive hypomineralized-hypoplastic |
| 38 | *ORAI1* | ORAI calcium release-activated calcium modulator 1 | 12q24.31 | Immunodeficiency 9 | autosomal-recessive hypocalcified |
| 39 | *PCNT* | pericentrin | 21q22.3 | Microcephalic osteodysplastic primordial dwarfism type II (MOPDII) | autosomal-recessive (no detailed information is available) |
| 40 | *PEX1* | peroxisomal biogenesis factor 1 | 7q21.2 | Neonatal adrenoleukodystrophy (NALD), Infantile Refsum disease (IRD), Zellweger syndrome, Heimler syndrome | autosomal-recessive hypoplastic, autosomal-recessive hypomineralized |
| 41 | *PEX6* | peroxisomal biogenesis factor 6 | 6p21.1 | Heimler syndrome | autosomal-recessive hypoplastic |
| 42 | *PEX26* | peroxisomal biogenesis factor 26 | 22q11.21 | Heimler syndrome | autosomal-recessive hypoplastic |
| 43 | *PHEX* | phosphate regulating endopeptidase homolog X-linked | Xp22.11 | X-linked hypophosphataemic rickets (XLHR) | X-linked hypoplastic |
| 44 | *RELT* | RELT TNF receptor | 11q13.4 | Isolated | autosomal-recessive hypomineralized |
| 45 | *ROGDI* | rogdi atypical leucine zipper | 16p13.3 | Kohlschutler-Tönz syndrome (KTS) | autosomal-recessive hypoplastic and/or hypomineralized/hypomaturation |
| 46 | *RUNX1* | runt related transcriptional factor 1 | 21q22.12 | Braddock-Cary syndrome (BCS) | autosomal hypoplastic |
| 47 | *RUNX2* | RUNX family transcription factor 2 | 6p21.1 | Metaphyseal dysplasia with maxillary hypoplasia and brachydactyly (MDMHB) | autosomal-dominant hypomineralized |
| 48 | *SLC4A1* | solute carrier family 4 member 1 (Diego blood group) | 17q21.31 | Distal renal tubular acidosis (dRTA) | autosomal-dominant dental defect (no detailed information is available) |
| 49 | *SLC4A4* (aka *NBCe1-A)* | solute carrier family 4 member 4 | 4q13.3 | Proximal renal tubular acidosis (pRTA) | autosomal-recessive hypoplastic |
| 50 | *SLC10A7* | solute carrier family 10 member 7 | 4q31.22 | Skeletal dysplasia with multiple dislocation, Congenital disorders of glycosylation (CDG) | autosomal-recessive hypoplastic/hypomineralized |
| 51 | *SLC13A5* | solute carrier family 13 member 5 | 17p13.1 | Kohlschutler-Tönz syndrome (KTS) | autosomal-recessive hypoplastic |
| 52 | *SLC24A4* | solute carrier family 24 member 4 | 14q32.12 | Isolated | autosomal-recessive hypomaturation/hypomineralized |
| 53 | *STIM1* | stromal interaction molecule 1 | 11p15.4 | Immunodeficiency 10 | autosomal-recessive hypomaturation |
| 54 | *TP63* | tumor protein p63 | 3q28 | Split hand-split foot-ectodermal dysplasia | autosomal-recessive hypoplastic/hypocalcified/hypomaturation |
| 55 | *VDR* | vitamin D receptor | 12q13.11 | Familial vitamin-D dependent rickets type 2A (VDDR2A) | autosomal-recessive hypoplastic |
| 56 | *WDR72* | WD repeat domain 72 | 15q21.3 | Distal renal tubular acidosis (dRTA), Isolated | autosomal-recessive hypomaturation |
| 57 | 21q22 microdeletion including RUNX1 coding region |  | 21q22 | Microdeletion syndrome (thrombocytopenia, mental/growth retardation, microcephaly etc.) | hypoplastic |
