# Supplementary material for: Overexpression of miR-1306-5p, miR-3195, and miR-3914 Inhibits Ameloblast Differentiation through Suppression of Genes Associated with Human Amelogenesis Imperfecta
Source: Int J Mol Sci. 2021 Feb 23;22(4):2202. doi: 10.3390/ijms22042202 (PMC7926528; doi:10.3390/ijms22042202)
Supplement: Supplementary file 1 [file ijms-22-02202-s001.zip › Supplementary Table S3_Final_021921.docx]

**Supplementary Table S3.** Syndromic amelogenesis imperfecta (AI) and enamel hypoplasia

| **No** | **Syndrome** | **Type of enamel defects** | **Suggested gene mutations** |
| --- | --- | --- | --- |
| 1 | Aarskog-Scott syndrome (AAS) | X-linked enamel hypoplastic AI | mutation in *FGD1* |
| 2 | Acro-dermato-ungual-lacrimal-tooth (ADULT) syndrome | enamel hypoplasia | mutation in *TP63* |
| 3 | ADOC Wang’s syndrome | enamel hypoplasia |  |
| 4 | AI with nephrocalcinosis (enamel-renal syndrome or McGibbon syndrome) | autosomal-recessive hypoplastic or hypomineralized AI |  |
| 5 | Alagille syndrome | enamel hypoplasia | mutation in *JAG2* in 90%, deletion of Chr20p (including *JAG2*) in 7%, and mutation in *NOTCH2* in a few patients |
| 6 | Alström syndrome | enamel hypoplasia | autosomal-recessive; mutation in *ALMS1* |
| 7 | Autosomal-recessive ectodermal dysplasia syndrome | enamel hypoplasia | mutation in *GRHL2* |
| 8 | Brachyolmia-platyspondyly-AI | hypoplastic AI |  |
| 9 | Celiac disease | enamel hypoplasia |  |
| 10 | Chondrodysplasia Grebe type | enamel hypoplasia | mutation in *GDF5* |
| 11 | Christ-Siemens-Touraine syndrome (CST) [Hypohidrotic ectodermal dysplasia (HED)] | enamel hypoplasia | mutation in *EDA* |
| 12 | Cleidocranial dysostosis (CCD) | enamel hypoplasia |  |
| 13 | Cockayne syndrome (CS) | enamel hypoplasia | mutations in *CSA* or *CSB* |
| 14 | Congenital adrenal hyperplasia (CAH) | autosomal-recessive hypoplastic AI | more than 95% of patients have a mutation in *CYP21A2* |
| 15 | Congenital chloride diarrhea (CCD) | hypoplastic and hypocalcified enamel | mutation in *SLC26A3* |
| 16 | Congenital contractural arachnodactyly (CCA) | enamel hypoplasia and hypomineralization | mutations in *FBN2* and *FBN1* are reported with an autosomal-dominant inheritance manner |
| 17 | Costello syndrome (CS) | hypomineralized or hypoplastic AI | mutation in *HRAS* |
| 18 | Cardiofaciocutaneous (CFC) syndrome | enamel defect | mutations in *BRAF* (75-80%), *MAP2K1*, *MAP2K2*, or *KRAS* are reported. |
| 19 | Camurati-Engelmann disease (diaphysial dysplasia) | enamel hypoplasia | autosomal-dominant; mutation in *TGFB1* |
| 20 | Cystic fibrosis (CF) | autosomal-recessive hypoplastic AI | mutation in *CFTR* |
| 21 | DiGeorge syndrome (22q11.2 deletion syndrome) | enamel hypoplasia and/or enamel hypocalcification | deletion in 22q11.2 |
| 22 | Distal renal tubular acidosis (dRTA) | hypoplastic AI | mutations in *ATP6V1B1*, *ATP6V0A4*, and *SLC4A1* are identified in primary dRTA patients |
| 23 | Dysosteosclerosis | enamel hypoplasia | autosomal-recessive; mutation in *SLC29A33*. |
| 24 | Ectodermal dysplasia syndrome | hypoplastic-hypocalcified enamel |  |
| 25 | Ectodermal dysplasia trichoodontoonychial type | enamel hypoplasia | maybe autosomal-recessive |
| 26 | Ectrodactyly-ectodermal dysplasia-clefting (EEC) syndrome 3 | enamel hypoplasia | mutation in *TP63* |
| 27 | Ekman-Westborg-Julin trait | enamel hypoplasia |  |
| 28 | Ellis-van Creveld syndrome (EvCS) | hypoplastic AI | mutations in *EVC* or *EVC2* |
| 29 | Enamel dysplasia with hamartomatous atypical follicular hyperplasia (EDHFH) | hypoplastic AI | unique disease in Black South African |
| 30 | Epidermolysis bullosa acquisita (EBA) | enamel hypoplasia |  |
| 31 | Epidermolysis bullosa atrophicans-gravis Herlitz | enamel hypoplasia | autosomal-recessive |
| 32 | Epidermolysis bullosa dystrophica (EBD) | enamel hypoplasia with hypomineralization | autosomal-recessive or dominant mutations in *COL7A1* |
| 33 | Epidermolysis bullosa simplex (EBS) | enamel hypoplasia | mutation in *ITGB4* |
| 34 | Epidermolysis bullosa simplex-Weber-Cockayne (EBS-WC) | enamel hypoplasia |  |
| 35 | Anderson-Fabry disease (angiokeratoma corporis diffusum: ACD) | hypoplastic AI |  |
| 36 | Familial hypophosphataemic vitamin D-resistant rickets (VDRR) | hypomineralized, or hypoplastic AI | mutations in *PHEX* (X-linked), *CLCN5*, *DMP1*, *ENPP1*, *FGF23*, and *SLC34A3* are identified |
| 37 | Fanconi syndrome (FS) | hypomineralized AI |  |
| 38 | Focal dermal hypoplasia (FDH) (Goltz-Gorlin syndrome) | hypoplastic AI | X-linked mutation in *PORCN* |
| 39 | Hallermann-Streiff syndrome (HSS) | enamel hypoplasia |  |
| 40 | Hamamy syndrome | enamel hypoplasia |  |
| 41 | Hypophosphatasia (dominant type) | enamel hypoplasia | autosomal-dominant; mutation in *ALPL* (*TNSALP)* |
| 42 | Idiopathic hypoparathyroidism | enamel hypoplasia |  |
| 43 | Inflammatory linear verrucous epidermal nevus (ILVEN) syndrome | enamel hypoplasia |  |
| 44 | Intestinal lymphangiectasia (IL) | enamel hypoplasia |  |
| 45 | Kartagener’s syndrome (KS) | linear enamel hypoplasia |  |
| 46 | KBG syndrome | enamel hypoplasia | mutation in *ANKRD11* or deletion of 16q24.3 |
| 47 | Keratosis follicularis spinulosa decalvans (KFSD) | enamel hypoplasia | either X-linked or autosomal-dominant |
| 48 | Krabbe disease | enamel hypoplasia | mutation in *GALC* |
| 49 | Lacrimo-auriculo-dento-digital (LADD) syndrome | enamel hypoplasia |  |
| 50 | Laryngo-onycho-cutaneous syndrome (LOCS) (Shabbir syndrome) | enamel hypoplasia | autosomal-recessive; mutation in *LAMA3* |
| 51 | Lenz-Majewski syndrome (LMS) | enamel hypoplasia | mutation in *PTDSS1* |
| 52 | Malignant osteopetrosis (MO) | enamel hypoplasia | mutations in *ATP6i* and *CLCN7* with autosomal recessive inheritance |
| 53 | Marfan syndrome | localized hypoplastic or hypomineralized, or generalized enamel defect | mutation in *FBN1* |
| 54 | Menkes syndrome | enamel defect | X-linked; mutation in *ATP7A* |
| 55 | Methylmalonic acidemias (MMAs) | enamel hypoplasia | around 50% of patient have mutations in *MUT* |
| 56 | Morquio syndrome (mucopolysaccharidosis type IVA) | autosomal-recessive hypoplastic AI | mutation in *GALNS* |
| 57 | Odontodysplasia | hypoplastic AI |  |
| 58 | Oculocerebrorenal syndrome of Lowe (OCRL) (Lowe syndrome) | enamel hypoplasia | mutation in *OCRL* |
| 59 | Osteogenesis imperfecta (OI) type III | hypomaturation AI | mutations in *COL1A1* or *COL1A2*. dentinogenesis imperfecta (DI) is frequently found in OI patients |
| 60 | Otodental syndrome (Otodental dysplasia) | enamel hypoplasia | autosomal-dominant basis |
| 61 | Papillon-Lefevre syndrome (PLS) | enamel hypoplasia | autosomal-recessive; mutation in *CTSC*. |
| 62 | Perrault syndrome | autosomal-recessive AI |  |
| 63 | PHACE syndrome | enamel hypoplasia |  |
| 64 | Phenylketonuria | enamel developmental defect |  |
| 65 | Prader-Willi syndrome (PWS) | enamel hypoplasia | deletion of paternal Chr15 or maternal uniparental disomy is reported |
| 66 | Proteus syndrome | enamel hypoplasia | mosaicism mutations in *AKT1* |
| 67 | Proximal renal tubular acidosis (pRTA) | enamel hypoplasia |  |
| 68 | Prune belly syndrome (PBS) | enamel hypoplastic and generalized hypocalcification |  |
| 69 | Pseudohypoparathyroidism | enamel hypoplasia |  |
| 70 | Pseudoxanthoma elasticum (PXE) | autosomal-recessive hypoplastic AI | mutation in *ABCC6* |
| 71 | Pycnodysostosis | enamel hypoplasia | mutation in *CTSK* |
| 72 | Ring chromosome 14 syndrome with trisomy X | enamel pit |  |
| 73 | Rothmund-Thomson syndrome (RTS) | enamel hypoplasia | autosomal-recessive; mutations in *RECQL4* in 2/3 patients and unknown in 1/3 patients |
| 74 | Rubinstein-Taybi syndrome (RSTS) | enamel hypoplasia | mutations in *CREBBP* (60%), *EP300* (10%), or unknown (30%) |
| 75 | Sanjad-Sakati syndrome | enamel hypoplasia |  |
| 76 | SCARF syndrome | enamel hypoplasia | X-linked recessive |
| 77 | Seckel syndrome (SCKL) | enamel hypoplasia | mutations in 10 genes (e.g. *ATR*, *CENPJ*, *CDK5RAP2,* etc.) |
| 78 | Silver-Russell syndrome | enamel defect | Chr7 and Chr11 |
| 79 | Sjögren-Larsson syndrome (SLS) | enamel hypoplasia | mutation in *ALDH3A2* |
| 80 | Smith-Magenis syndrome (SMS) | enamel hypoplasia | 90% is a 17p11.2 deletion (affecting *RAI1*), and 10% is a heterozygous mutation in *RAI1* |
| 81 | Sotos syndrome | enamel hypoplasia | mutation in *NSD1* or deletion of 5q35 |
| 82 | Spondylocarpotarsal synostosis syndrome (SCT) | enamel hypoplasia | mutation in *FLNB*. *MYH3*, or *RFLNA* |
| 83 | Syndrome of facial, oral and digital anomalies | enamel hypoplasia | trisomy of 7q21.2-a22.1 |
| 84 | Syndrome of mild mental retardation, early onset of baldness, and enamel hypoplasia | enamel hypoplasia | microduplication of *NF1* |
| 85 | Thurston syndrome (Orofaciodigital syndrome type V) | enamel hypoplasia |  |
| 86 | Trisomy 9p | enamel hypoplasia |  |
| 87 | Tuberous sclerosis (TS) | enamel hypoplasia or pitted enamel | autosomal-dominant; mutations in *TSC1* or *TSC2* |
| 88 | Turner syndrome | enamel hypoplasia | 45X, aberrations of chromosome X, or mosaic karyotype |
| 89 | Usher syndrome (USH) type II | autosomal-recessive hypoplastic AI | at least three causative loci identified (1q41, 3p23-24, and 5q) |
| 90 | Williams syndrome (elfin facies syndrome) | enamel hypoplasia | autosomal-dominant; deletion of Chr7 |
